# The impact of ageing on health care expenditures: a study of steepening

**DOI:** 10.1007/s10198-013-0541-9

**Published:** 2013-11-24

**Authors:** Fredrik Alexander Gregersen

**Affiliations:** 1 Health Services Research Unit, Akershus University Hospital, Boks 95, 1478 Lørenskog, Norway; 2 Institute of Clinical Medicine, Campus Akershus University Hospital, University of Oslo, Oslo, Norway

**Keywords:** *Red herring hypothesis*, Hospital expenditure, Trends in health care expenditures, *Steepening*, Ageing, A19, I15, I19

## Abstract

Some researchers claim that health care expenditures for older people are growing faster than for the rest of the population. This process is referred to as *steepening*. The aim of this paper is to test *steepening*, applying new data and revised methods. Furthermore, we explain the connection between the terms *red herring hypothesis*, i.e., that time to death and not age per se drives the health care expenditures, and *steepening*. We also present the mechanisms that may induce *steepening*, as presented in the literature. When testing *steepening*, we apply data from all inpatient stays in somatic hospitals in Norway in the period 1998–2009, i.e., the data has no self-selection and covers the entire population of Norway (5 million). Our analysis does not reject *steepening*, with the exception of the 0-year-olds. The results also hold when controlling for mortality-related expenditures. Furthermore, we observe an increase in expenditures for the 0-year-olds. Finally, we find increasing mortality-related expenditures over time. We find the link between *steepening* and the *red herring hypothesis* to be vague, and we find *steepening* and the *red herring hypothesis* to be independent.

## Introduction

The proportion of the elderly population in the countries of the Organization for Economic Co-operation and Development (OECD) will increase substantially in the coming years. Knowledge regarding the distribution of per capita health care costs between different age groups is essential in order to forecast future health care expenditures. In this study, we discuss the long-term development of health care expenditures. Even though the long-term developments for the entire population are discussed in detail in several papers [1, 2], the specific increase for different age groups is poorly covered. This is vital in order to understand the long-term developments in health care expenditures. In this paper, we will investigate the specific growth for different age groups.

When discussing trends in health care expenditures, two concepts are often brought up in the literature: *red herring hypothesis* and *steepening*. The *red herring hypothesis* states that health care expenditures are driven by time to death, not age per se [3]. *Steepening* states that the growth in per capita health care expenditures for older people is higher than for the rest of the population [4]. In this paper, we will focus on the latter, but clarify the relation between the terms. The aim of the clarification is threefold. First, in the literature [4, 5], the connection between *steepening* and the *red herring* is vaguely described, so a clarification will therefore contribute to the *steepening* literature. Second, a discussion of the link between the terms will contribute to further understanding of the concept of *steepening*. Third, several methodological issues discussed in the *red herring* debate also apply in the *steepening* debate, and hence bringing in the *red herring* literature will improve the *steepening* debate.

The aim of this paper is threefold. First, we measure changes in health care expenditures over time, in order to test if *steepening* may be rejected. Second, we present mechanisms that may induce *steepening*, as presented in the literature. Third, we attempt explain the connection between *red herring* and *steepening*.

When testing *steepening*, we apply a complete data set for inpatient hospital expenditures in Norway from 1998 to 2009. We use diagnostic related groups (DRG) weights to measure the hospital expenditures. Norway has a National Health Service similar to the one we find in other Scandinavian countries and the United Kingdom (UK). The hospitals are public and financed through general taxation [6].

The contribution of this paper is that we elaborate on the link between *red herring* and *steepening* more than has previously been done in the literature [4, 5]. We also summarize the literature on mechanisms that may induce *steepening*. Furthermore, the estimation techniques previously presented in the literature are improved and the previous methods are replicated. The data set applied to test *steepening* is of high quality, with no self-selection over a long period of time.

The paper proceeds as follows: first, we present the terms *steepening* and *red herring* with the present literature, and discuss in detail what may induce *steepening*. Secondly, we explain the link between the two concepts. Thirdly, we test *steepening*. In the third part, we first present the data, then the methods and the results. Fourth, we present the conclusion and discussion.

## Background

“If steepening [occurs]…, the future increase of health care costs will even be larger than in the predictions which keep expenditure profiles constant”[4] p 582.

From the quote above, *steepening* may be seen as a contradiction to the more optimistic future scenarios described in the *red herring* debate [2], which claim that future health care expenditures will be lower than previously expected, due to an increased length of life. However, as we will return to in the end of this section, both hypotheses may in fact hold at the same time. Before the link between the terms is explained in more detail, we will summarize the literature on the *steepening* and briefly mention the *red herring* literature.

### Steepening

In 2006, a new term regarding health care expenditures and older people was introduced by Buchner and Wasem [4] that suggested per capita health care expenditures would grow faster for the elderly than for younger people, i.e., a situation characterized by *steepening*. *Steepening* was defined as the increase in the ratio of per capita expenditures for older people (65+) divided by the younger (below 65), over time:

1 $$\overline{Y}_{a \in [65,106],t} /\overline{Y}_{a \in [0,64],t} > \overline{Y}_{a \in [65,106],t - 1} /\overline{Y}_{a \in [0,64],t - 1}$$

where $\overline{Y}_{a \in [65,106],t}$ is the per capita expenditures for the elderly (aged above 65+) in year *t*.

Note that in their regression analysis they defined the young to be between 30 and 64, while the old were between 65 and 79 [4]. To make the age limits more comparable with the other definition (2) of *steepening* presented in this paper, we will use the age limits as presented in definition (1) throughout this paper. Also note that Buchner and Wasem [4] include other definitions of *steepening* that we will return to in the “Methods” section.

Based on the same definition, but without using the term *steepening*, health data from OECD between 1984 and 1998 indicates *steepening* in several countries, among them the United States (US), Finland and Japan. However, this pattern is not found in the UK [7], where a decline in the expenditures for the elderly compared to the rest of the population is observed. There are also other studies that suggest health care expenditures grow faster for the elderly than the rest of the population [8–11]. There are, however, methodological issues connected to the simple method (definition) used in these papers, which we will discuss in more detail later. Some of the methodological issues are solved by Felder and Werblow [5], who defined *steepening* as a positive cross derivative of per capita health care expenditures with respect to age and time:

2 $$\frac{{\partial^{2} \overline{Y}_{g} (a,t)}}{\partial a \partial t} > 0.$$

Note that Felder and Werblow [5] included mortality rates in the function of per capita expenditures, in contradiction with the definition by Bucher and Wasem [4], as they defined *steepening* in three dimensions (age, per capita expenditures and time). Therefore, in the rest of this section we will ignore the impact of mortalities.

Definition (2) forms the basis of this paper, but results based on both definitions (1) and (2) will be presented later. The reason for focusing on the latter definition is that the definition is more flexible with respect to model specification, and in our view it captures the concept as it was originally formulated by Bucher and Wasem [4]. A wider discussion on the different definitions of *steepening* will be presented in the methods section.

Felder and Werblow [5] mention several factors that may lead to *steepening* or reduce the effect of *steepening*. We will give a short summary in the following section. They suggest that *steepening* may arise due to increased “maintenance” costs as length of life increases, or simply as a bias in the technological frontier (more innovations in medical treatments for older people). They also mentioned that, to the contrary, per capita mortality-related expenditures for hospitals are decreasing with age; hence, increased length of life might reduce mortality-related expenditures. This is supported in several studies [12–14]. Felder and Werblow [5] also suggest that, due to compression of morbidity, the period of illness will be compressed over time, which will in turn reduce the per capita health care expenditures related to older people [15].

Another paper discussing the reasons for growth in health care expenditures for the elderly is written by Barer et al. [11]. They discuss the implications of changes in morbidity and mortality and how that might change utilization for health care. Their study is formed around rectangularization of survival curves over time, compression of mortality [16], and compression of morbidity [15]. They argue that based on the preferences of society to either accept “natural death” or use all resources possible to reduce morbidity, the compression of mortality and morbidity will influence health care expenditures in different ways. If society accepted “natural death”, health care expenditures for the elderly will drop over time, while if society minimizes morbidity it will increase expenditures for elderly.

In summary, the literature on the causes of increased expenditures for the elderly indicates that there might be a technological bias and changes in biological factors (morbidity). With regard to the first, the technological bias is likely to be driven by some underlying mechanisms that are poorly explained by Felder and Werblow [5]. One reason might be biological changes over time, but there could also be other mechanisms driving *steepening*.

### Red herring

The *red herring hypothesis* was formulated by Zweifel et al. [3], and states that health care expenditures are driven by time to death and not age per se. A similar idea had previously been presented by Fuchs [17]. Zweifel et al. [3] formulated precisely as:

The health care expenditures for an individual (*i*) independent of age:

3 $$\frac{{\partial Y_{i} (a,k)}}{\partial a} = 0$$

where *a* is age and *k* is quarters to death.

The health care expenditures are dependent on quarters to death:

4 $$\frac{{\partial Y_{i} (a,k)}}{\partial k} \ne 0.$$

Several studies have tested the *red herring hypothesis* (see, among others, [2, 18, 19]); i.e., the studies have tested how time to death and age for a sample of the population may explain the observed health care expenditures. Some of the studies reject, while other support, the *red herring hypothesis*.

In the *red herring* debate, several methodological problems have been raised (see. among others. [20, 21]). The debate is summarized in Häkkinen et al. [22] by pointing at two econometrical issues: first, multicollinearity between the explanatory variables (age and time to death), and second, endogeneity between health care expenditure and time to death (mortalities). Both these issues will be relevant in the “Methods” section in Eqs. 10, 11, 14 and 16. Gregersen and Godager [13] apply the same data set as we do in this study, and discuss both these issues in detail. In summary, first, the multicollinearity is of minor importance, as the data set is large; second, the assumption that mortalities are exogenous is not rejected.

### The link between steepening and red herring

By definition, *steepening* is defined in three dimensions (age, time, and per capita health care expenditures) as is the *red herring* (age, time to death and individual health care expenditures). As the dimensions in the terms differ with respect to time and time to death, the link between the terms is not obvious, and both hypotheses may hold at once. Furthermore, when comparing (2) (*steepening*) with (3) and (4) (*red herring*), the definitions of the terms do not contradict or support each other. In summary, we therefore conclude that the terms are independent.

## Data

For this study, we have repeated cross-sectional data (pseudo-panel) for all hospital admissions in Norway from 1998 until 2009. The data comes from the Norwegian Patient Registry (NPR). The data was merged with demographic characteristics from Statistics Norway (SSB). The data from NPR provides a complete registry of all hospital admissions in Norway from January 1998 to December 2009. The dataset contains data on somatic in patient care. Registration in NPR is compulsory for all hospitals, and therefore there is no self-selection in the dataset. Each admission to the hospital (hospital stay) is registered as an observation, and it is not possible to track individuals between admissions. The dataset contains five variables; year of birth, gender, year of hospital stay, DRG-points (diagnostic related group) and place of residence of the patient (municipality). Data on the number of inhabitants (N) are given by SSB (Table 1).

**Table 1:** Descriptive statistics: expenditures

Variable	Number of observations	Mean	Std. dev.	Min	Max
Per capita expenditures by year: $\overline{Y}_{t} = \frac{1}{{N_{t} }}\sum\limits_{i \in t} {Y_{i} }$	12	7,340.71	947.5119	5,786.21	8,595.46
Per capita expenditures by group: $\overline{Y}_{g} = \frac{1}{{N_{g} }}\sum\limits_{i \in g} {Y_{i} }$	995,158	10,453.61	17,145.92	0	1,006,657

In order to get per capita measures, we aggregated the data by grouping the data so the smallest possible cell is defined by a given age ($a_{i}$), gender ($q_{i}$), year ($t_{i}$) and municipality ($m_{i}$). The 430 municipalities, 106 ages, 2 genders and years of observation (1998–2009) gave 1,093,920 unique cells that form the dataset our analysis is based on. We index the cells with the index *g* (*g* = 1, 2…,1,093,920).

The per capita expenditure in one cell is:

5 $$\overline{Y}_{g} = \frac{1}{{N_{g} }}\sum\limits_{i \in g} {Y_{i} }$$

The per capita hospital expenditures in year (*t*) are defined by:

6 $$\overline{Y}_{t} = \frac{1}{{N_{t} }}\sum\limits_{i \in t} {Y_{i} }$$

**Fig. 1:**
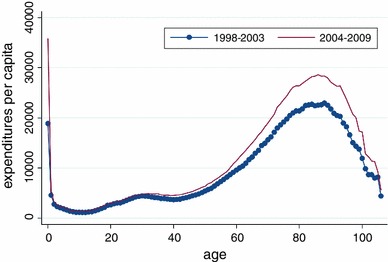
Hospital expenditures per capita measured in NOK over age

For the rest of this paper, the expenditure will be measured in Norwegian kroner (NOK), inflation adjusted to 2010 NOK {8 NOK = 1 € [Norwegian Bank (2010)]}. In Fig. 1 we present per capita expenditures as a function of age. To explore how expenditures have developed over time for different age groups, we compared the per capita expenditures for the first 6 years with the last 6 years in the dataset. We aggregate the total health care expenditures for each age (*a*) and divide by the number of inhabitants with age (*a*), for each of the two time periods (1998–2003 and 2004–2009). If we denote the start of a period by *t*_1_ and the end by *t*_2_ (for example *t*_1_ = 1998 and *t*_2_ = 2003) the health care expenditures for age (*a*) in Fig. 1 is defined by:

7 $$\overline{Y}_{{a,t \in \left[ {t_{1,} t_{2} } \right]}} = \frac{1}{{N_{{a,t \in \left[ {t_{1} ,t_{2} } \right]}} }}\sum\limits_{{i \in \left\{ {a,t \in \left[ {t_{1} ,t_{2} } \right]} \right\}}} {Y_{i} }.$$

The figure clearly shows that the per capita health care expenditures for the older people and newborns (0 years of age) have increased substantially over time during the period of observation. Except for newborns, the expenditures for older people increased more than for the rest of the population. If newborns are excluded, the figure shows that the expenditures for older people have grown faster than for the rest of the population, consistent with *steepening*.

One reason for the increase in expenditures for newborns may be increased expenditures for premature infants. Both Bratlid and Nordermoen [23] and Nordermoen and Bratlid [24] discusses the increases in treatment expenditures for treatment of premature infants in Norway. In summary, they highlight that more premature infants with low birth weight are treated, and advances in technology not only increase the cost of treatment, but also improve the quality of the treatment.

Table 2 compares demographic characteristics for the first 6 years (1998–2003) in the data set with the last 6 years (2004–2009). Comparing the two periods, the average annual number of inhabitants has increased from 4.5 million in the first period to 4.7 million in the last period. The total number of decedents does decline over time, comparing the same two periods from 263,627 to 249,902. The mortality rate (number of decedents divided by the total population) for most age groups is falling over time, apart from the age groups containing the individuals aged between 5 and 14.

**Table 2:** Descriptive statistics: demographic characteristics

Age	2004–2009			1998–2003			Mortality rate 1–mortality rate 2
	#Decedents	Inhabitants	Mortality rate 1	#Decedents	Inhabitants	Mortality rate 2	
0	943	351,791	0.002681	1,146	342,773	0.003343	−0.0007
1–4	359	1,390,934	0.000258	531	1,420,461	0.000374	−0.0001
5–9	192	1,795,174	0.000107	231	1,827,422	0.000126	0.0000
10–14	186	1,868,604	0.0001	222	1,752,313	0.000127	0.0000
15–19	607	1,834,645	0.000331	718	1,591,994	0.000451	−0.0001
20–24	995	1,681,558	0.000592	1,168	1,634,271	0.000715	−0.0001
25–29	1,173	1,728,377	0.000679	1,378	1,886,462	0.00073	−0.0001
30–34	1,252	1,932,635	0.000648	1,681	2,077,068	0.000809	−0.0002
35–39	1,791	2,130,113	0.000841	2,084	2,000,640	0.001042	−0.0002
40–44	2,416	2,066,621	0.001169	2773	1,903,477	0.001457	−0.0003
45–49	3,699	1,931,564	0.001915	4,049	1,835,113	0.002206	−0.0003
50–54	5,741	1,847,509	0.003107	6,505	1,807,816	0.003598	−0.0005
55–59	8,617	1,776,023	0.004852	8,690	1,550,439	0.005605	−0.0008
60–64	12,527	1,586,827	0.007894	10395	1,148,655	0.00905	−0.0012
65–69	14,342	1,137,507	0.012608	14,833	1,004,696	0.014764	−0.0022
70–74	18,734	925,269	0.020247	24,233	989,335	0.024494	−0.0042
75–79	29,838	848,292	0.035174	39,216	941232	0.041,665	−0.0065
80–84	45,382	725,662	0.062539	50,893	716,681	0.071012	−0.0085
85–89	51,417	475,017	0.10824	49,216	405,885	0.12126	−0.0130
90+	49,691	244,942	0.20287	43,665	200,843	0.21741	−0.0145
Sum	249,902	28,279,064		263,627	27,037,576		−0.053386
Average sum by year (sum/6)	41,650	4,713,177		43,938	4,506,263		

## Methods; identifying steeping

In Buchner and Wasem [4], three methods are presented with which to identify *steepening*. The first is based on definition (1); using this approach, they find clear evidence of *steepening*. As they state, the clear advantage of this simple method is that it is transparent and easy to replicate. On the other hand it does not investigate changes within the two age groups—the younger and older people. This is closely related to:

8 $$\overline{Y}_{a \in [65,106],t} /\overline{Y}_{a \in [0,64],t} = \psi_{0} + \psi_{1} *t.$$

Second, they suggest a slightly modified method, using a benchmark age group, and compare the growth of the other age groups relative to the benchmark age group:

9 $$\frac{{\overline{Y}_{a,t} }}{{\overline{Y}_{{{\text{benchmark}},t}} }} = \lambda_{0,a} + \lambda_{1,a} *t.$$

Finally, they suggested a model with health care expenditures as an exponential function of age. Buchner and Wasem [4] only had 20 age groups and two genders, each year for 18 years (20 × 18 × 2 = 720 observations). The data limitations put strong limitations on their regression methods. The methods were later significantly improved by Felder and Werblow [5]. They had more variation in the data (a larger data set) with 26 regions, both genders, 10 years and 20 age groups, i.e., 10,400 observations. This allowed for a more complex model. They assumed that the health care expenditures are a function of time (*t*) and demographics [age (*a*), gender (*q*) and mortality rate $(\phi )$]:

10 $$\begin{gathered} {\text{Per capita health care expenditure }} \hfill \\ \quad = {\text{Constant}} + \beta *{\text{gender}} + \gamma *{\text{age}} + \theta *{\text{time}} + \kappa *{\text{mortality rate}} + \mu *{\text{age}}*{\text{time}} + {\text{error term}} \hfill \\ \quad \leftrightarrow \hfill \\ \end{gathered}$$

11 $$\overline{Y}_{g} = \alpha_{{}} + \beta *q_{{}} + \gamma *a + \theta *t + \kappa *\phi_{g} + \mu *a*t + \varepsilon_{g}.$$

In Eqs. 10 and 11,^1^ $\beta$ indicates the marginal increase in cost for females compared to males, and $\theta$ captures yearly growth in per capita expenditures, while $\mu$ measures the age specific growth rate as deviation from the yearly growth rate ($\theta$), $\kappa$ is the increase in per capita hospital expenditures due to mortality rate, and $\gamma$ is the impact of age on per capita expenditures. Finally, $\varepsilon$ is the error term. In this setting, *steepening* was defined by (2).

Note that Felder and Werblow [5] argue that hospital expenditures grow exponentially over time. To test if an exponential or linear model applies to our data set, we ran two regressions: first, keeping the dependent variable as a linear function of time, and second, keeping the dependent variable as an exponential function of time:

12 $$\overline{Y}_{g} = \theta_{0} + \theta_{1} *t$$

with *R* ^2^ = 0.0095 and $\ln \overline{Y}_{g} = \theta_{0} + \theta_{1} *t$ with *R* ^2^ = 0.0019.

From Eq. 12 we see that the *R* ^2^ is low in both the exponential and linear model, but slightly higher in the linear model. Based on the result, the difference between the two models is small and both models may apply. However, we choose to apply a linear model due to the slightly higher *R* ^2^.

Felder and Werblow [5], argue that $\frac{{\partial^{2} \overline{Y} }}{{\partial a_{{}} \partial t}}$ is a function not only of $\mu_{a}$, but also of the mortality rate $\phi$. They assume that $\frac{{\partial^{2} \phi }}{{\partial a_{{}} \partial t}} < 0$ due to increased length of life. Therefore, we tested the magnitude of changes in mortality rate from changes in age and time:

13 $$\phi_{g} = \alpha_{0} + \alpha_{1} *t*a + \alpha_{2} *a + \alpha_{3} *t + \varepsilon_{g}.$$

In the rest of the methods section, we will ignore the impact of changes in mortalities on *steepening*, but we will come back to this issue in the results section.

Further, as stated earlier, Felder and Werblow [5] only had 20 age groups ($d_{z}$) in their data set, limiting their analysis to 14. As we have more variation in the age variable, we are not forced to keep the same grouping of the regression parameter. However, per capita expenditure is not a linear function of age (see Fig. 1); therefore, we also treat age as a categorical variable, with 21 groups, respectively. The reasons for keeping age to 21 groups only are twofold. First, it will make the results easier to compare to the methods presented by Felder and Werblow [5]. Second, if age is treated with one-year age-groups, the number of observations in each group declines, and therefore the precision of each estimate will drop. In summary, as the estimate of interest here is the differences in growth between the young and old, and not the specific growth rate for each age per se, we therefore find the grouping similar to the one found in Felder and Werblow [5] to be sensible in this analysis:

14 $$\begin{aligned} \overline{Y}_{g} & = \alpha + \beta *q + \sum\limits_{z = 0}^{20} {\gamma_{z} *d_{z} + \theta *t + \sum\limits_{x = 1}^{4} {\kappa_{x} } *(\phi_{g} )^{x} } \\ \quad + \sum\limits_{z = 11}^{20} {\mu_{z} d_{z} *t + \varepsilon_{g} } \\ \end{aligned}$$

$d_{z}$ is a dummy for indicating age group (0, 1–4, 5–9, …, 90+), $\varepsilon_{m,a,t,q}$ represents the error term, and $\mu_{z}$ measures the deviation in growth rate for age group z compared to the young [below 50 (*z* < 11)]. As $\mu_{{z \in \{ 1,10\} }}$ is the benchmark age group, *steepening* is for age group *z* as defined by:

$\mu_{z} - \mu_{{z \in \{ 1,10\} }} > 0$ for z > 10, indicating that the growth rate for the elderly is higher than for the young.

*Steepening* within the 50+ age group is defined by $\mu_{z + 1} - \mu_{z} > 0$ for *z* > 10.

The reasoning for choosing the specific functional form to capture the mortality-related expenditures in Eq. 14 is poorly described by Felder and Werblow [5]. From several papers [12–14], it is known that mortality-related health care expenditures are a decreasing function of age. We therefore include the interaction between age and mortalities $({\text{age}}*\phi )$ in our analysis. Furthermore, we cannot find any studies supporting the inclusion of mortalities to the power of two, three and four (*x* = 2, 3, 4). We therefore choose to only include mortality rate to the power of one (*x* = 1). The number of mortalities, due to compression of morbidity, increases for the highest age groups (see Table 2). We would therefore expect, as mortality related expenditures decrease with age, to observe a reduction in the mortality related expenditures over time. To capture the latter effect, we include the interaction between mortalities and time, which we expect to be negative:

15 $$\frac{{\partial^{2} \overline{Y}_{g} }}{\partial \phi \partial t} = \lambda < 0.$$

Finally, we also include the yearly growth rate for all age groups ($\mu_{z}$, *z* = 0, 1, ..., 20), to identify differences within the young. We are now left with the equation that forms the basis of our analysis:

16 $$\begin{aligned} \overline{Y}_{g} & = \alpha + \beta *q + \sum\limits_{z = 0}^{20} {\gamma_{z} *d_{z} + \theta *t + \kappa *\phi_{g} } + \eta *\phi_{g} *a \\ \quad + \sum\limits_{z = 0}^{20} {\mu_{z} *d_{z} *t + \lambda *\phi_{g} *t + \varepsilon_{g} } \\ \end{aligned}.$$

We note that the error terms in Eqs. 10–16 are heteroscedastic, due to variation in the size of the cells, $N_{g}$. We therefore weight the regressions by the number of inhabitants in each cell.

## Results

This section will present estimations based on the methods presented in methods section. The share of the per capita health care expenditures used by the elderly (65+) does not increase over time (1998–2009) (Table 3). This holds even though we exclude the newborns. On the contrary, the share used by the younger group is highest in 1998. The estimation based on Eq. 1, therefore, does not support *steepening*. When running a regression on Table 3, equivalent to (8), we find a negative and significant effect when including all ages $\psi_{1} = - 0.021$. Furthermore, when excluding individuals <1 year of age, we find a positive, not significant effect $\psi_{1} = 0.003$. Overall, the estimation effect based on (1) and (8) rejects *steepening*.

**Table 3:** The share of total health care expenditures spent on the elderly compared to the rest of the population

Year	Including all ages $\overline{Y}_{a \in [65,106],t} /\overline{Y}_{a \in [0,64],t}$	Excluding age zero $\overline{Y}_{a \in [65,106],t} /\overline{Y}_{a \in [1,64],t}$
1998	4.524	4.760
1999	4.204	4.346
2000	4.290	4.442
2001	4.334	4.483
2002	4.117	4.452
2003	4.113	4.431
2004	4.143	4.496
2005	4.164	4.510
2006	4.098	4.447
2007	4.159	4.567
2008	4.107	4.490
2009	4.239	4.648

To identify *steepening* in Eqs. 10, 11, 14 and 16, the magnitude of the changes in mortality over time has to be identified. As discussed in the methods section, mortality rates are decreasing over time, i.e., there is a compression of mortalities (see Table 2). From the regression on (13), we find the effect to be small, significant, and negative ($\alpha_{1} = - 0.0000085$) (see Table 4). To also estimate the effect of changes in mortality rates over time in (14), i.e., mortality rate to the power of 1, 2, 3 and 4, we also included regressions with the mortalities to the power of 2, 3, and 4 as the dependent variable in Table 4.

**Table 4:** Results from regression analysis based on Eq. 13

Dependent variable	Mortality rate $\left( \phi \right)$		$\left( {\phi^{2} } \right)$		$\left( {\phi^{3} } \right)$		$\left( {\phi^{4} } \right)$	
Independent variable	Coefficient	Std. er.	Coefficient	Std. er.	Coefficient	Std. er.	Coefficient	Std. er.
Age	0.000708***	(−0.00000256)	0.000126***	(−0.00000139)	0.0000502***	(−0.00000121)	0.0000327***	(−0.00000116)
Time	0.000129***	(−0.0000176)	0.0000274**	(−0.00000957)	0.0000106	(−0.0000083)	0.00000661	(−0.00000798)
Age × time	−0.00000850***	(−0.00000039)	−0.00000149***	(−0.000000212)	−0.000000546**	(−0.000000184)	−0.000000336	(0.00000017)
Constant	−0.0169***	(−0.000115)	−0.00339***	(−0.0000626)	−0.00140***	(−0.0000543)	−0.000922***	(−0.0000522)
*N*	995,158		995,158		995,158		995,158	
*R* ^2^	0.194		0.025		0.005		0.003	

Table 5 presents four regressions. The first is based on Eq. 14 in the “Methods” section. As expected, the age coefficient for the younger age group is low (below 25), apart from the 0-year-olds. The age coefficient peaks for the 70–75-year-olds. For the highest age groups, there is a decline compared with the age group 70–75. We may not reject *steepening* in this model based on the analysis:

17 $$\begin{aligned} \frac{{\partial^{2} \overline{Y} }}{\partial a\partial t} = & \mu + \frac{{\partial^{2} \sum\nolimits_{x = 1}^{4} {\kappa_{x} \left( \phi \right)^{x} } }}{\partial a\partial t} = \mu_{z} - \mu_{{z \in \left\{ {1,10} \right\}}} + \frac{{\partial^{2} \sum\nolimits_{x = 1}^{4} {\kappa_{x} \left( \phi \right)^{x} } }}{\partial a\partial t} = \mu_{z} - \mu_{{z \in \left\{ {1,10} \right\}}} \\ \quad + 131415.8*( - 0.0000085) + ( - 446127.2)*( - 0.00000147) \\ \quad + 640520.0*(0.000000546) + ( - 310920.1)*( - 0.000000336) > 0 \\ \end{aligned}.$$

**Table 5:** Results from regression analysis based on Eqs. 14 and 16

Dependent variable per capita expenditures $(\overline{Y}_{g} )$								
Equation	(14)		(16) Excluding mortalities		(16) Excluding the interaction between time and mortalities		(16) Including the interaction between time and mortalities	
Independent variable	Coefficient	Standard er.	Coefficient	Standard er.	Coefficient	Standard er.	Coefficient	Standard er.
Year (*t*) *t* = 0 if year = 1998 *t* = 1 if year = 1999 … *t* = 11 if year = 2009 $(\theta )$	142.1***	(2.024)						
*t* × age $(\mu )$								
*t* × age 0			2,700.4***	(14.75)	2,731.0***	(14.39)	2,725.1***	(14.38)
*t* × age 1–4			76.86***	(7.365)	82.03***	(7.184)	81.43***	(7.181)
*t* × age 5–9			38.12***	(6.545)	38.74***	(6.384)	38.52***	(6.381)
*t* × age 10–14			44.18***	(6.598)	45.24***	(6.436)	45.02***	(6.433)
*t* × age 15–19			58.88***	(6.699)	62.99***	(6.534)	62.24***	(6.531)
*t* × age 20–24			53.80***	(6.751)	58.96***	(6.584)	57.71***	(6.581)
*t* × age 25–29			56.33***	(6.454)	58.06***	(6.295)	56.65***	(6.292)
*t* × age 30–34			97.25***	(6.250)	101.9***	(6.096)	100.4***	(6.093)
*t* × age 35–39			130.4***	(6.152)	136.8***	(6.001)	135.0***	(5.999)
*t* × age 40–44			147.7***	(6.202)	155.7***	(6.049)	153.1***	(6.047)
*t* × age 45–49			164.5***	(6.401)	173.0***	(6.243)	168.9***	(6.242)
*t* × age 50–54	51.53***	(6.710)	183.3***	(6.454)	195.5***	(6.295)	188.6***	(6.296)
*t* × age 55–59	115.2***	(7.236)	243.0***	(7.008)	259.7***	(6.836)	249.3***	(6.842)
*t* × age 60–64	247.9***	(7.741)	371.8***	(7.537)	393.0***	(7.352)	375.7***	(7.372)
*t* × age 65–69	368.6***	(8.509)	476.6***	(8.336)	509.2***	(8.132)	481.4***	(8.182)
*t* × age 70–74	552.0***	(9.058)	625.7***	(8.901)	682.4***	(8.685)	636.8***	(8.816)
*t* × age 75–79	741.8***	(9.417)	790.2***	(9.268)	862.5***	(9.045)	783.7***	(9.421)
*t* × age 80–84	819.3***	(10.61)	867.9***	(10.49)	948.9***	(10.24)	811.9***	(11.23)
*t* × age 85–89	866.5***	(13.28)	939.4***	(13.22)	1,021.8***	(12.90)	787.4***	(15.12)
*t* × age 90+	778.4***	(18.61)	913.5***	(18.64)	976.4***	(18.19)	548.5***	(23.19)
Gender $(q)$	197.4***	(11.55)	−92.59***	(11.58)	222.7***	(11.38)	222.7***	(11.37)
Age $(\gamma )$								
0	23,927.5***	(57.03)	9,705.0***	(107.8)	8,761.0***	(105.3)	8,783.9***	(105.3)
1–4	Reference		Reference		Reference		Reference	
5–9	−1,554.6***	(33.81)	−1,368.4***	(63.77)	−1,283.1***	(62.20)	−1,285.0***	(62.18)
10–14	−1,847.8***	(33.81)	−1,678.6***	(64.59)	−1,592.9***	(63.00)	−1,594.7***	(62.97)
15–19	−1,093.4***	(34.23)	−962.8***	(65.62)	−960.5***	(64.00)	−959.7***	(63.97)
20–24	−80.42*	(34.49)	96.26	(65.16)	35.33	(63.55)	38.47	(63.52)
25–29	956.1***	(33.83)	1,108.1***	(63.06)	1,063.5***	(61.51)	1,067.2***	(61.48)
30–34	1,368.6***	(33.09)	1,306.3***	(62.16)	1,252.2***	(60.63)	1,256.5***	(60.61)
35–39	1,011.4***	(32.89)	799.1***	(62.62)	706.4***	(61.08)	713.0***	(61.05)
40–44	985.8***	(33.17)	722.4***	(63.06)	569.6***	(61.51)	580.1***	(61.48)
45–49	1,760.5***	(33.55)	1,496.4***	(63.69)	1,240.6***	(62.13)	1,258.7***	(62.10)
50–54	2,807.8***	(50.25)	2,885.2***	(63.74)	2,453.2***	(62.20)	2,486.0***	(62.19)
55–59	4,405.1***	(54.24)	4,731.7***	(66.91)	4,076.6***	(65.34)	4,127.6***	(65.33)
60–64	6,089.4***	(59.61)	6,813.5***	(71.24)	5,831.2***	(69.64)	5,919.5***	(69.68)
65–69	8,264.0***	(62.92)	9,649.5***	(73.68)	8,200.5***	(72.18)	8,347.5***	(72.31)
70–74	10,326.0***	(64.89)	12,811.1***	(74.42)	10,679.8***	(73.21)	10,929.5***	(73.66)
75–79	11,538.5***	(68.44)	15,688.1***	(75.63)	12,646.0***	(74.99)	13,081.9***	(76.37)
80–84	10,803.2***	(79.57)	17,176.7***	(83.18)	13,006.6***	(83.41)	13,767.3***	(87.20)
85–89	8,676.3***	(101.8)	17,490.2***	(101.3)	12,186.2***	(103.3)	13,499.3***	(112.3)
90+	4,508.2***	(141.9)	14,703.5***	(136.1)	9,302.3***	(145.1)	11,712.1***	(166.1)
Mortality rate $(\kappa )$	131,415.8***	(871.7)			297,704.7***	(1,759.7)	289,556.8***	(1,780.1)
Mortality rate^2^	−446,127.2***	(7,069.6)						
Mortality rate^3^	640,520.0***	(16,487.0)						
Mortality rate^4^	−310,920.1***	(10,454.6)						
Age × mortality rate $(\eta )$					−2,932.4***	(19.91)	−2,966.2***	(19.93)
Mortality rate × *t* $(\lambda )$							2,045.9***	(68.79)
Constant $(\alpha )$	2,053.2***	(32.60)	2,881.3***	(50.74)	2,291.4***	(49.57)	2,294.2***	(49.55)
*N*	995,158		995,158		995,158		995,158	
R^2^	0.605		0.598		0.618		0.618	
Adjusted R^2^	0.605		0.598		0.618		0.618	

Within the group of older people (above 50), we find *steepening* for all age groups $(\mu_{z + 1} - \mu_{z} > 0)$ apart from the highest age group, above 90 $(\mu_{20} - \mu_{19} < 0)$. Furthermore, the effect of mortalities^2^ $\left( {\frac{{\partial \overline{Y}_{g} }}{\partial \phi } > 0} \right)$ is positive, and females have higher expenditures than males on average.

Second, the regression output based on Eq. 16 excludes mortalities. In this regression, the 0-year-olds have the highest yearly growth, of 2,700 NOK $\left( {\mu_{0} = 2700.4} \right)$. The second-highest yearly growth is found for the 80–85-year-olds, with 939.4. In comparison, the 5–9-year-olds have a yearly growth rate of 38.12. The annual growth for young individuals (i.e., below age 50) is lower than for those individuals age 50 or greater, that is, apart from the newborns. This does not reject *steepening* if newborns are excluded.

Third, the regression output based on Eq. 16 is presented, but now including the effect of mortalities, while excluding the interaction between time and mortalities. As expected, the effect of mortalities is positive, and as expected the mortality related cost is a decreasing function of age. Also, the age effect is slightly reduced here for each age group, implying that part of the expenditures for each age group is generated by mortalities. Especially for the highest age groups, there is a decline from a model excluding mortalities. The yearly growth rate for the different age groups are similar to the previous (second) results presented, and the same interpretation regarding *steepening* applies.

Finally, the results from running a regression on Eq. 16 both including the effect of the interaction of mortalities and time are presented. When the interaction of time and mortalities are included, the yearly growth rate is declining for all age groups, apart from the 1–4 group. The yearly growth for the 90+ was 976.4; after the inclusion of the interaction term it became 548.5, implying that part of the growth for the highest age groups is caused by increased mortality related costs over time $(\lambda = 2045.9)$.

To summarize the results in Table 5, the first regression does not reject *steepening* (based on Eq. 14). In the following three regressions presented (based on Eq. 16), we can also not reject *steepening* if excluding individuals below age 1.

## Conclusion and discussion

The first part of this paper clarified the connection between *steepening* and the *red herring hypothesis*. We concluded that the terms are independent. Furthermore, the data applied in this study is insufficient to test the *red herring hypothesis*. The reason for the data “insufficiency” is that the data do not contain information on time-to-death at the individual level. Therefore, the data may not reject the hypothesis as formulated by Zweifel et al. [3], i.e. (3) and (4).

The first part of this paper continued with summarizing causes mentioned in the literature that may induce *steepening*. In summary, the literature is limited and points at biological and technological factors.

The second part of this paper was to test *steepening*. *Steepening* was defined by Buchner and Wasem [4] in three dimensions: time, age and per capita health care expenditures. In these dimensions, the term states that health care expenditures should grow faster for older people than the rest of the population. In these dimensions, we find evidence of *steepening* with the exception of the 0-year-olds, i.e., Eq. 16, excluding mortalities. The method is similar to the method found in Felder and Werblow [5]. When using definitions (1) and (8), similar to the methods suggested by Buchner and Wasem [4], we find no evidence of *steepening*, including all ages. However, when excluding the individuals aged zero, we find a non-significant effect in (8) in favour of *steepening*. Our results are not directly comparable to Buchner and Wasem [4], as they only included individuals between 30 and 70 years of age in their study. Regardless of the age limits used, the latter model has little flexibility within the age groups (young and older), as there is only one dummy for each group. From Fig. 1, it is clear that per capita health care expenditures is not a linear function of age, and a model allowing for more variation is more appropriate. Overall, we therefore find the results based on Eq. 16, excluding mortalities, to be more reliable.

The second step in our empirical estimations was then to estimate what factors may drive the *steepening* effect. From several studies, among them Zweifel et al. [3] and Seshamani and Gray [25], mortalities are an important driver of health care expenditures. We would therefore expect the effect of *steepening* to be reduced in Eq. 16, including mortalities. In Table 5, it is shown that such a decrease does not occur. However, when including the interaction between mortalities and time, the *steepening* effect strongly declines, i.e., part of the *steepening* effect is driven by increased mortality-related expenditures over time.

Several implications follow from the results. First, as shown in several other studies (see, among others, [12, 26]) both mortality and age contribute to health care expenditures. Second, per capita health care expenditures are biased towards older individuals over time. Per capita health care expenditures for infants are increasing more than for the rest of the younger population. Third, if the observed trend continues, expenditures for older individuals are likely to increase substantially in the future (both due to increased expenditures towards elderly in general and increased expenditures for decedents [based on Table 5, last regression]). However, the implication of the results with regard to predictions of future health care expenditures should be interpreted with care until to the mechanisms that drive *steepening* are detected.

The only health care service included in this study is inpatient in somatic hospitals; this is a limitation to this study. If there is a substitution effect between different health care services, excluding other services could potentially lead to biased results. It may be plausible that *steepening* is observed for inpatients, but the opposite effect is observed in other health care services. Additional research should therefore take place in other parts of the health care sector in order to confirm *steepening* outside inpatient care.

The use of DRG-cost weights to measure expenditure enables the study to investigate costs for different age and gender groups over time. There are, however, some limitations associated with using DRGs as a proxy for costs. DRG-cost weights are the expected cost of a treatment for the average patient and not the actual cost. As mentioned by Melberg et al. [12], elderly individuals have poorer health than the average patient, and the cost for this group might therefore be underestimated. Conversely, for other healthier groups, the use of DRG-weights may have overestimated actual costs.

In summary, our results clearly do not reject *steepening* in per capita health care expenditures over time for the 50+ age group, with the exception of 0-year-olds. Mortality-related expenditures also increase over time, and the effect of *steepening* is reduced when this effect is taken into account.

## Footnotes

1 Note that g denotes a cell in the data set applied in this study, characterized by age, gender, time and municipality.

2
$$\begin{aligned} \frac{{\partial \overline{Y}_{g} }}{{\partial \phi_{g} }} & = \kappa_{1} + 2\kappa_{2} \phi_{g} + 3\kappa_{3} \phi_{g}^{2} + 4\kappa_{4} \phi_{g}^{3} = 131416 - 2*446127*\phi_{g} \\ & \quad + 3*640520*\phi_{g}^{2} - 4*310920*\phi_{g}^{3} > 0\forall \phi_{g}. \\ \end{aligned}$$
